# Alpha-1 Antitrypsin Reduces Severity of *Pseudomonas* Pneumonia in Mice and Inhibits Epithelial Barrier Disruption and *Pseudomonas* Invasion of Respiratory Epithelial Cells

**DOI:** 10.3389/fpubh.2013.00019

**Published:** 2013-06-21

**Authors:** Gregory B. Pott, K. Scott Beard, Courtney L. Bryan, Daniel T. Merrick, Leland Shapiro

**Affiliations:** ^1^Denver Veterans Affairs Medical Center, Denver, CO, USA; ^2^University of Colorado Anschutz Medical Campus, Aurora, CO, USA

**Keywords:** pneumonia, alpha-1 antitrypsin, cytokines, sepsis, *Pseudomonas*, neutrophil elastase

## Abstract

Nosocomial pneumonia (NP) is the third most common hospital-acquired infection and the leading cause of death due to hospital-acquired infection in the US. During pneumonia and non-pneumonia severe illness, respiratory tract secretions become enriched with the serine protease neutrophil elastase (NE). Several NE activities promote onset and severity of NP. NE in the airways causes proteolytic tissue damage, augments inflammation, may promote invasion of respiratory epithelium by bacteria, and disrupts respiratory epithelial barrier function. These NE activities culminate in enhanced bacterial replication, impaired gas exchange, fluid intrusion into the airways, and loss of bacterial containment that can result in bacteremia. Therefore, neutralizing NE activity may reduce the frequency and severity of NP. We evaluated human alpha-1 antitrypsin (AAT), the prototype endogenous NE inhibitor, as a suppressor of bacterial pneumonia and pneumonia-related pathogenesis. In AAT^+/+^ transgenic mice that express human AAT in lungs, mortality due to *Pseudomonas aeruginosa* (*P.aer*) pneumonia was reduced 90% compared to non-transgenic control animals. Exogenous human AAT given to non-transgenic mice also significantly reduced *P.aer* pneumonia mortality. *P.aer*-infected AAT^+/+^ mice demonstrated reduced lung tissue damage, decreased bacterial concentrations in lungs and blood, and diminished circulating cytokine concentrations compared to infected non-transgenic mice. *In vitro*, AAT suppressed *P.aer* internalization into respiratory epithelial cells and inhibited NE or *P.aer*-induced disruption of epithelial cell barrier function. The beneficial effects of human AAT in murine *P.aer* pneumonia raise the possibility of AAT use as a prophylactic treatment for NP in humans, and suggest a role for AAT as an innate immune mediator.

## Introduction

Pneumonia is often categorized by location of acquisition. Community-acquired pneumonia (CAP) is contracted in the non-health care setting, and nosocomial pneumonia (NP) is acquired in the hospital. NP is of special interest due to a high prevalence in hospitalized patients, a poor prognosis, and a large expenditure of resources. NP is the third most common hospital-acquired infection in the US (1–3), with about 250,000 cases of NP in 2002 (3). NP is the leading cause of death due to hospital-acquired infection (2, 3), and in 2002 there were nearly 36,000 NP-related deaths in the US (crude mortality of about 15%) (3). NP is caused predominantly by Gram-negative rods (nearly 42% of isolates), with *Pseudomonas aeruginosa* (*P.aer*) accounting for about 20% of isolates (2, 4, 5).

Alarmingly, mortality due to lower respiratory tract infection was not diminished over 25 years of observation in the US (6). Attributable mortality is especially high for *P.aer* NP that is acquired while receiving mechanical ventilation, reported at 43% (1). Bacteria that cause NP have demonstrated escalating resistance to antibiotics over time (5). Unfortunately, the current antibiotic pipeline has slowed and emergence of more potent antimicrobial drugs is unlikely to address NP-related therapeutic challenges (7). NP carries substantial resource burdens that include annual total treatment costs in the US nearing $5.4 billion (3, 8). These considerations indicate that NP is a substantial clinical problem, and recent treatments and preventative (prophylactic) measures have shown little clinical impact (9).

The pathogenesis of pneumonia usually involves bacterial colonization of the upper airways followed by aspiration of these bacteria into the lower respiratory tract (4, 10, 11). Pneumonia develops when bacteria and secretions aspirated into the lower airways is sufficient to overcome lower respiratory tract host defenses. Lower levels of aspirated bacteria may cause pneumonia during illness that weakens host defenses. Bacterial components induce inflammation by stimulating alveolar macrophages and respiratory epithelial cells to produce pro-inflammatory cytokines such as interleukin (IL)-1, tumor necrosis factor (TNF)α, and the chemokine IL-8 (12, 13). IL-8 recruits and activates neutrophils, which secrete neutrophil elastase (NE) into the respiratory tract. Clinical studies have demonstrated substantial NE levels in bronchoalveolar lavage fluid (BALF) in patients with *P.aer* NP (14, 15).

Neutrophil elastase activities in the airways promote pneumonia severity (12). Since NE is an omnivorous protease, NE can directly damage lung epithelial cells and supporting tissues. This results in reduced capacity to eliminate bacteria, defective gas exchange, and exudation of fluid into lung airspaces (12, 16, 17). NE induces production of pro-inflammatory cytokines in the lungs that augment inflammation (16, 18, 19), and NE inactivates several extracellular immune mediators such as immunoglobulins, complement components, and cathelicidin (16, 20, 21). NE also induces expression of MUC1 (a cell-surface mucin) that can serve as a receptor for *P.aer* and perhaps other bacteria (22, 23). Since bacteria can spontaneously invade respiratory epithelium (22, 24–26), NE-enhanced binding of bacteria to respiratory epithelial cells likely initiates invasion. Translocation of bacteria into the cell interior permits evasion from extracellular antimicrobial substances such as antibodies, complement, lysozyme, lactoferrin, cathelicidin-related molecules, and defensins (13, 27). Intracellular translocation also sequesters bacteria from the antibacterial activities of macrophages and neutrophils. Since NE can also increase vascular permeability, NE may enhance translocation of bacteria across the endothelial cell barrier and initiate bacteremia (12). During established pneumonia, these NE activities in the lower respiratory tract amplify lung inflammation and tissue damage (12). NE may also promote bacterial proliferation and bacteremia during pneumonia. Elevated lower respiratory tract NE has also been demonstrated during non-pneumonia systemic illness. This may result in NE-induced defects in host defense that link underlying systemic disease to increased NP risk (28–30).

Since excessive NE activity in the respiratory tract participates in pneumonia pathogenesis, NE inhibition is a target for therapeutic intervention. Alpha-1 antitrypsin (AAT) is the prototype endogenous inhibitor of serine proteases such as NE. AAT is a 394 amino acid, 52 kDa glycoprotein produced primarily by the liver and secreted into the circulation. AAT is the most abundant endogenous serine protease inhibitor in the circulation, with serum concentrations reported as 1.0–2.7 mg/mL in healthy adults (31). AAT is an acute phase protein (32), and circulating concentrations can increase two to fourfold during systemic inflammation (33, 34). AAT concentrations in lung tissues are substantially less than concentrations in the circulation, with extracellular lung fluid levels of about 10% serum concentrations (35). The function of AAT is classically described as neutralization of NE in the lung in order to limit NE-induced tissue damage (36). There are over 100 AAT variants in humans, and clinical interest in AAT has focused on genetic AAT deficiency caused by inheritance of two copies of the abnormal Z-type AAT gene. Z-type AAT is characterized by defective export from the liver into the circulation, and serum AAT levels are reduced to 10–15% of normal (37). The best-described clinical consequences of AAT deficiency include pulmonary emphysema and liver disease (37). Pulmonary emphysema is thought to originate from an imbalance between protease (NE) and antiprotease (AAT) that favors protease destruction of lung tissue (37). The only specific treatment for AAT deficiency is replacement therapy with intravenous AAT purified from healthy donor plasma.

In a prior report, AAT was used as a treatment for chronic bacterial pneumonia in rats (38). In that report, 7 days of aerosolized AAT was administered as a post-infection treatment in rats with *Pseudomonas* lung infection. AAT-treated rats demonstrated a delayed reduction in bacterial levels in lungs and reduced lung inflammation. We assessed a different approach to AAT use in pneumonia. In our studies, we tested the hypothesis that human AAT would provide preventative (prophylactic) protection against acute pneumonia, since AAT clinical use is likely optimal as a preventative treatment. Since human AAT neutralizes murine NE, human AAT can be used to examine NE suppression in mouse models (39). Therefore, we conducted mouse studies to examine the protective effect of AAT present before bacterial infection. Mortality, lung histopathology, bacterial quantification in the lungs and peripheral blood, and serum cytokine levels were determined. We also assessed mechanisms of AAT protection *in vitro*. AAT was examined for direct antibacterial activity, for effect on *P.aer* cell-surface binding and internalization into A549 human lung epithelial cells, and for effect on NE- or *P.aer*-induced disruption of A549 monolayer barrier integrity.

## Materials and Methods

### Cells and reagents

The gentamicin-sensitive PA01 *P.aer* bacterium was obtained from the American Type Culture Collection (ATCC, Manassas, VA, USA) and cultured in Luria–Bertani broth (LB, Fisher Scientific, Fair Lawn, NJ, USA) as suspension cultures or on LB-agar plates at 37°C. A549, a human lung alveolar epithelial cell line, was also obtained from the ATCC. A549 culture medium comprised RPMI 1640 medium supplemented with 10% heat-inactivated fetal bovine serum (Sigma-Aldrich, St. Louis, MO, USA), and 2 mM l-glutamine without or with 100 U/mL penicillin and 100 μg/mL streptomycin (all from Mediatech, Inc., Herndon, VA, USA). All A549 cell culture incubations were conducted in a 37°C and 5% CO_2_ atmosphere incubator. Clinical-grade AAT was obtained from Baxter (Aralast NP, Westlake Village, CA, USA). Human NE was obtained from Innovative Research (Novi, MI, USA). All cultures were analyzed for toxicity using a lactate dehydrogenase (LDH) release assay (Promega, Madison, WI, USA).

### Mouse studies

All procedures and care complied with the Institutional Animal Care and Use Committee at the Denver Veterans Affairs Medical Center. Female C57Bl/6 mice were purchased from The Jackson Laboratory (Bar Harbor, ME, USA). A transgenic AAT^+/+^ mouse strain containing a human AAT gene under control of the lung Surfactant Protein C promoter (40), was backcrossed onto a C57Bl/6 background and obtained from Eli Lewis, University of Colorado Denver (41).

### Mouse infection with *P.aer*

For all mouse infection experiments, 8- to 12-week-old female AAT^+/+^ transgenic mice or non-transgenic C57Bl/6 control mice were anesthetized with isoflurane (Vedco, Inc., St. Joseph, MO, USA) and infected by nasal aspiration of a 50 μL suspension containing 1 × 10^6^ colony forming units (cfu) of viable *P.aer*. Body weights were measured twice daily, and moribund mice (body weight loss>20%) were euthanized. In experiments examining the effect of exogenous AAT on *P.aer* pneumonia, C57Bl/6 non-transgenic mice received three 30 min treatments of aerosolized AAT or sterile saline (control) using a nebulizer (nebulizer obtained from Mabis Healthcare, Waukegan, IL, USA) at 48, 24, and 2 h before *P.aer* nasal aspiration. The nebulization rate was 0.2 mL/min and the total AAT dose was 120 mg per treatment. In addition, mice were given three intraperitoneal (ip) injections with either 200 μL (2 mg) AAT or 200 μL saline (control) at 48, 24, and 2 h before infection with *P.aer*.

### Histopathological assessment of lung involvement in mice infected with *P.aer*

Alpha-1 antitrypsin^+/+^ transgenic mice and C57Bl/6 non-transgenic control mice were infected by nasal inhalation of 1 × 10^6^ cfu of viable *P.aer*. Twenty-four hours after infection, mice were anesthetized with Nembutal (Oak Pharmaceuticals, Lake Forest, IL, USA) and the lungs were inflated *in situ* with 4% paraformaldehyde in phosphate buffered saline (PBS) (pH 7.4) to fix the tissues. The lungs were excised and stored at 4°C overnight in 4% paraformaldehyde. After 24 h, the lungs were washed in 5.0 mL PBS, embedded in paraffin, and sectioned. Hematoxylin and eosin (H&E) staining was performed at National Jewish Health Core Laboratory (Denver, CO, USA). Whole mount sections of both lungs from each mouse were evaluated by a single examiner (DTM) blinded to experimental condition. Pneumonia was classified as lobar pneumonia or bronchopneumonia in each lung lobe according to the extent of inflammation in each of the five lobes. If the large majority of a lobe (>80%) showed evidence of inflammation, involvement was defined as lobar. Bronchopneumonia in a lobe was defined by the presence of distinct peri-bronchial inflammation that involved<80% of the lung tissue. All lung sections were also examined for alveolar hemorrhage and the number of lobes with hemorrhage was counted for each mouse (usually five lung lobes per mouse). In a second analysis, the percent of each lobe occupied by inflammatory infiltrate was estimated. An overall percent of lung inflammation for each mouse was calculated as the mean percent involvement in all lobes in the two lungs. The presence and severity of necrosis was also examined and subjectively reported as early or advanced.

### Quantification of *P.aer* in mouse lungs and peripheral blood

C57Bl/6 control and AAT^+/+^ mice were infected by nasal inhalation of 1 × 10^6^ cfu of *P.aer*. Twenty-four hours after infection, mice were euthanized and both lungs excised and weighed. Lungs were then homogenized in 1.0 mL PBS using a Tissue Tearor homogenizer (BioSpec Products, Bartlesville, OK, USA). Lung homogenates were diluted 1:100 into 1.0 mL LB medium, 200 μL aliquots of this dilution spread onto duplicate LB-agar plates, and the plates incubated at 37°C for 18 h. After counting the number of colonies, the number of bacteria per gram of lung tissue was calculated by multiplying the average number of colonies per agar plate by 500 and then dividing this value by the total lung weight to obtain the number of colonies per gram lung tissue.

For quantification of *P.aer* in blood, peripheral blood was obtained via heart puncture under sterile conditions from the same mice used for lung bacterial cultures. One milliliter of cardiac blood was transferred into a sterile tube containing 1.0 mL of BacT/ALERT SA blood culture medium (bioMérieux, Inc., Durham, NC, USA). Two hundred microliter aliquots of this dilution were spread onto duplicate LB-agar plates, and the plates were incubated at 37°C for 18 h. The bacterial colonies on the plates were counted, and the average number of colonies per plate was multiplied by 10 to obtain the number of *P.aer* bacteria in 1.0 mL blood.

### Cytokine measurements in mouse serum

Alpha-1 antitrypsin^+/+^ transgenic mice and C57Bl/6 non-transgenic control mice were infected by nasal inhalation of 1 × 10^6^ cfu of *P.aer*. Twenty-four hours after infection, the mice were euthanized and peripheral blood obtained via heart puncture. The blood was collected in BD Microtainer tubes containing lithium heparin (BD), and centrifuged at 2000 × *g* for 3 min. The supernatant plasma was transferred to a fresh tube and stored at −70°C until cytokine quantification. Mouse IL-1α, IL-1β, IL-2, IL-3, IL-4, IL-5, IL-6, IL-10, IL-12, IL-17, MCP-1, IFNγ, TNFα, MIP-1α, GMCSF, and RANTES were measured using a multiplex array chemiluminescence device (Quansys Biosciences, Logan, UT, USA).

### *P.aer* bacterial growth in liquid suspension cultures

Separate 2.0 mL aliquots of LB broth were inoculated with 2 × 10^6^ cfu of *P.aer*, one without and one with 5 mg/mL AAT, and the cultures were placed in a shaking incubator at 37°C. Culture aliquots were obtained during each hour of incubation for 8 h. The culture optical density (OD) at 600 nm was obtained as a measure of bacterial content (38).

### *P.aer* adherence and internalization into A549 cells

For bacterial adherence experiments, 1 × 10^6^ A549 cells were added into wells in a 24-well polystyrene tissue culture plate and incubated for 24 h in antibiotic-free culture medium. After 24 h, A549 cells adhered to culture well bottom surfaces and the culture medium was replaced with 1.0 mL fresh antibiotic-free culture medium without or with 5 mg/mL AAT. For cells exposed to human NE, human NE (2.0 μg/mL final concentration) was added to wells 1 h after AAT addition. Following an additional 1 h incubation, 0.2 × 10^6^ cfu (0.2 multiplicity of infection, MOI) of viable log-phase *P.aer* were added to all wells. Following addition of *P.aer*, the cultures were incubated for 1 h at 37°C and 5% CO_2_. The supernatants were aspirated and the cells washed three times with PBS to remove bacteria that were not cell-associated. The cells were lysed by removing PBS and adding 0.5 mL of 0.25% Triton X-100 into each well for 10 min. The lysates were diluted 1:1000 with LB broth, 200 μL aliquots were spread onto duplicate LB-agar plates using a spinner disk, and the plates incubated at 37°C for 18 h to facilitate bacterial growth. After counting all bacterial colonies on the agar plates, the total number of colonies in culture wells was calculated by multiplying the average number of colonies per plate by 2500.

For bacterial internalization experiments, 1 × 10^6^ A549 cells were added into five wells in 24-well polystyrene tissue culture plates and incubated for 24 h in antibiotic-free culture medium. After 24 h, the A549 cells adhered to culture well bottom surfaces and the culture medium was replaced with 1.0 mL fresh antibiotic-free culture medium without or with 5 mg/mL AAT. For cultured cells exposed to human NE, human NE (2.0 μg/mL final concentration) was added to wells 1 h after AAT addition. Following an additional 1 h incubation, 0.2 × 10^6^ cfu (0.2 MOI) of viable log-phase *P.aer* were added to all wells. The cultures were incubated for 2 h at 37°C and 5% CO_2_, and the supernatants aspirated and the cells washed three times with PBS to remove bacteria that were not cell-associated. The PBS was aspirated, 1.0 mL of antibiotic-free culture medium supplemented with 200 μg/mL Gentamicin (Sigma-Aldrich) was added to the wells, and the cells incubated for an additional 2 h to kill non-internalized bacteria. Cells were then washed three times with PBS and lysed by removing the PBS and adding 0.5 mL of 0.25% Triton X-100 into each well for 10 min. The lysates were diluted 1:100 with LB broth, 200 μL aliquots were spread onto duplicate LB-agar plates using a spinner disk, and the plates incubated at 37°C for 18 h to facilitate bacterial growth. After counting all colonies on the agar plates, the total number of colonies in culture wells was calculated by multiplying the average number of colonies per plate by 250.

### Transepithelial electrical resistance (TEER) assay

Transepithelial electrical resistance (TEER) is used as a measure of barrier integrity of cell monolayers in culture, where electrical resistance across a cell monolayer is directly proportional to barrier integrity (42). TEER assay experiments were conducted using six-well polystyrene tissue culture plates, with each well containing a cell culture insert with bottom-surface 0.4 μm pores (Fisher Scientific). One million A549 cells were added to the pore-containing inserts (upper chambers) along with 2.5 mL antibiotic-free culture medium. Three milliliters of antibiotic-free culture medium was added to wells in the six-well plates (lower chambers) and the cultures incubated for 24 h. This produced confluent A549 monolayers that formed a barrier between the upper and lower chambers. The medium was aspirated from upper and lower chambers and replaced with fresh antibiotic-free culture medium without (medium control) or with 5 mg/mL AAT. For cells exposed to human NE, 2.0 μg/mL final concentration human NE was added to upper chambers 1 h after AAT addition. A 5.0 MOI (5 × 10^6^ cfu) of viable log-phase *P.aer* bacteria were added to the upper (insert) chambers of all culture wells except for medium-alone control wells. For TEER experiments conducted in the absence of *P.aer*, TEER measurements were performed at 1, 3, and 6 h following addition of NE, and for experiments conducted in the presence of *P.aer*, TEER measurements were performed at 24, 30, and 48 h using an EVOM resistance meter as instructed by the manufacturer (World Precision Instruments, Sarasota, FL, USA). Electrical resistance was also measured in an insert containing well in the absence of A549 cells (defined as electrical resistance = 0). This value for resistance was subtracted from all other measurements. For each experimental condition, a time 0 measurement (TEER measured just prior to addition of *P.aer*) was set to 100% and subsequent measurements were calculated as a portion of 100%.

### Statistical analysis

For mouse mortality experiments, results were calculated as percent survival and Kaplan–Meir mortality curves were generated. Statistical significance was assessed by the log-rank test (Prism by GraphPad, La Jolla, CA, USA). For bacterial quantification comparisons, the Mann–Whitney *U*-test was performed. For TEER assay studies and experiments comparing serum cytokine levels, differences between experimental conditions were evaluated using ANOVA repeated measures with Bonferroni’s comparison test. *p* < 0.05 was defined as statistically significant for all analyses.

## Results

### *P.aer* pneumonia mortality is reduced in AAT^+/+^ mice and in mice given exogenous human AAT

**Figure 1:**
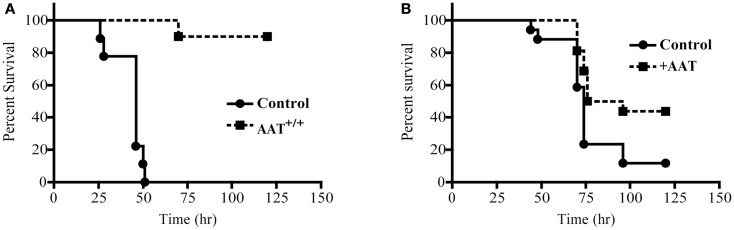
**Reduced mortality following *P.aer* pneumonia in AAT transgenic mice and in mice given exogenous AAT**. In **(A)** 10 transgenic mice that express human AAT in lung tissue (AAT^+/+^) and 9 C57Bl/6 (Control) mice were infected with inhaled *P.aer*. Kaplan–Meier survival curves are shown for 120 h following infection. Reduced mortality in AAT^+/+^ mice was statistically significant (*p* < 0.0001). In **(B)** C57Bl/6 mice were treated with exogenous AAT (16 mice, +AAT) or with saline (17 mice, Control). AAT was administered by nebulizer and intraperitoneal injection prior to infection with inhaled *P.aer*. Kaplan–Meier survival curves are shown for 120 h following infection. Reduced mortality in +AAT mice was statistically significant (*p* = 0.021).

Mortality was quantified following *P.aer* infection in transgenic mice that express human AAT (AAT^+/+^) and in C57Bl/6 (control) mice. As shown in Figure 1A, 10 AAT^+/+^ mice demonstrated significantly less pneumonia-related death (*p* < 0.0001) compared to 9 control mice, with 90% of the AAT^+/+^ mice surviving infection and 0% of control mice surviving infection. For the first 48 h, weight loss was comparable between the two groups (data not shown). After 48 h, surviving mice began to recover lost weight. In Figure 1B, exogenous human AAT was administered to 16 C57Bl/6 mice by both aerosol delivery using a nebulizer and ip injection. Seventeen control animals received PBS using the same two routes of administration. Forty-four percent of AAT-treated mice compared to 12% of control mice survived infection (*p* = 0.021).

### AAT^+/+^ mice demonstrate reduced lung damage following *P.aer* pneumonia

Six AAT^+/+^ mice and five C57Bl/6 non-transgenic (control) mice were infected with inhaled *P.aer*. Twenty-four hours after infection, lungs were fixed, sectioned, stained, and analyzed for pathological effects. One control animal had only four identifiable lung lobes. Table 1 displays blinded histological assessments of the number of lung lobes demonstrating lobar pneumonia, bronchopneumonia, or alveolar hemorrhage. The percent total lung involvement analysis describes the estimated percent of total lung tissue involved with pneumonia.

**Table 1:** **Lung histopathology in six AAT^+/^^+^ and five C57Bl/6 control mice infected with *P.aer***.

Mouse	Lobar pneumonia*	Broncho pneumonia*	Alveolar hemorrhage*	% total lung involved with inflammation	Necrosis
AAT^+/+^	0/5	5/5	0/5	35	No
AAT^+/+^	3/5	2/5	0/5	65	Yes-early
AAT^+/+^	2/5	3/5	0/5	55	Yes-early
AAT^+/+^	0/5	5/5	1/5	35	No
AAT^+/+^	0/5	5/5	0/5	45	No
AAT^+/+^	3/5	2/5	2/5	70	No
Total involvement	8/30 (27)^‡^	22/30 (73)^‡^	3/30 (10)^‡^	51^§^	NA
C57Bl/6	3/5	2/5	0/5	70	Yes-adv
C57Bl/6	3/5	2/5	3/5	85	Yes-adv
C57Bl/6	1/5	4/5	0/5	45	Yes-adv
C57Bl/6	2/4^†^	2/4^†^	4/4^†^	70	Yes-adv
C57Bl/6	5/5	0/5	5/5	100	Yes-adv
Total involvement	14/24 (58)^‡^	10/24 (42)^‡^	12/24 (50)^‡^	74^§^	NA

*NA, not applicable; Adv, advanced*.

*Fractions represent number of lung lobes with pathology compared to number of lung lobes examined.

*^†^Only four lobes out of five were identified*.

*^‡^Results shown as total number of lung lobes involved/total number of lung lobes examined (percent total involvement)*.

*^§^Mean percent total lung involvement for all animals in each group. *p* = 0.052 comparing these two groups*.

Reduced severity of pneumonia-associated inflammation was observed in AAT^+/+^ mice. A lobar pneumonia pattern was observed in 27% of AAT^+/+^ mouse lobes and in 58% of control mouse lobes. A bronchopneumonia pattern was observed in 73% of AAT^+/+^ lung lobes and in 42% of control lung lobes. These observations indicate reduced involvement in AAT^+/+^ mice, since bronchopneumonia is defined as less severe disease involvement than lobar pneumonia (see Materials and Methods). Alveolar hemorrhage was described in 10% of AAT^+/+^ mice and in 50% of control animals. The summary percent of total lung involved with inflammation showed less involvement in AAT^+/+^ mice (51%) compared to control animals (74%). Comparison of percent of total lung involvement in these two groups approached (but did not achieve) statistical significance (*p* = 0.052). Necrosis was observed in two of six AAT^+/+^ mice, with early stages of necrosis present in both. In contrast, necrosis was observed in five of five non-transgenic control animals, and in every case the necrosis was in advanced stages.

**Figure 2:**
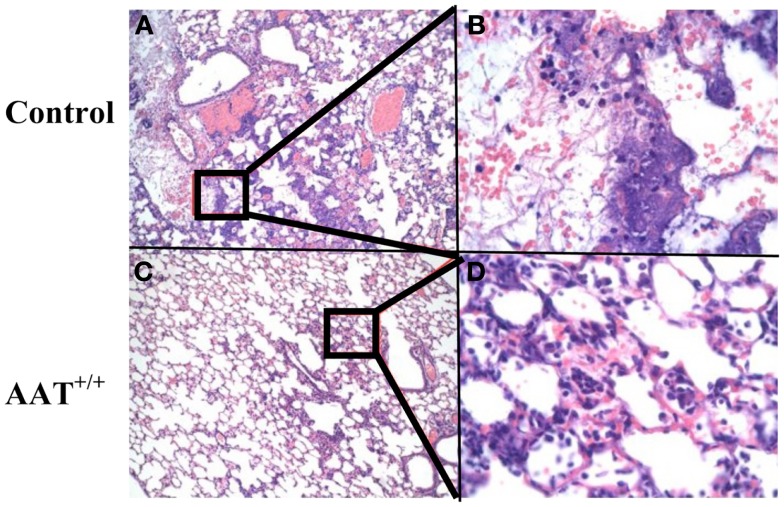
**Less inflammation in lungs of AAT transgenic mice infected with *P.aer***. Six transgenic (AAT^+/+^) mice and five C57Bl/6 non-transgenic control (Control) mice were infected with inhaled *P.aer*. Twenty-four hours after infection the mice were euthanized and lungs excised, fixed, sectioned, and stained with H&E. Representative photomicrographs of lung tissues are shown, with control lung sections in **(A,B)**, and AAT^+/+^ lung sections in **(C,D)**. Inset regions **(B,D)** are magnified views of the areas indicated in black rectangles in **(A,C)**, respectively. **(B,D)** show perivascular cuffing with predominant mononuclear infiltration that is common in bacterial pneumonia. Magnification is 20× for **(A,C)** and 200× for**(B,D)**.

Infected lung sections from representative control and AAT^+/+^ mice are presented in Figure 2. There is less pneumonia-associated inflammation in the AAT^+/+^ mouse lung compared to the control mouse lung, and decreased alveolar fluid extravasation and inflammatory cell infiltration in AAT^+/+^ lung (compare control lung tissue in Figures 2A,B to AAT^+/+^ lung tissue in Figures 2C,D).

### Reduced *P.aer* bacterial concentrations in lungs and blood in AAT^+/+^ mice

**Figure 3:**
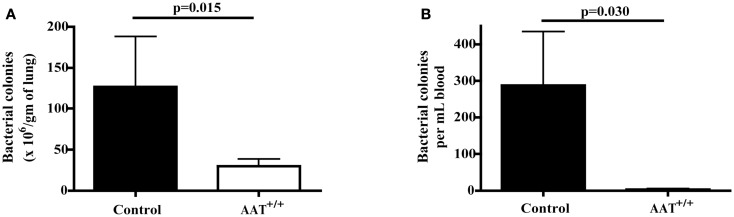
**Reduced bacterial concentrations in lungs and blood in AAT transgenic mice following *P.aer* pneumonia**. Six transgenic (AAT^+/+^) mice and six C57Bl/6 non-transgenic control (Control) mice were infected with inhaled *P.aer*. After 24 h, mice were euthanized and lung tissues and blood samples collected and processed as described in Section “Materials and Methods.” Bacterial concentrations were quantified in lungs **(A)** and in blood **(B)**. Graphs depict means + SEM and *p*-values are indicated in the graphs.

Six AAT^+/+^ mice and six non-transgenic control mice were infected with inhaled *P.aer*. Twenty-four hours following infection, lungs were removed and homogenized and peripheral blood was obtained via heart puncture. Compared to controls, AAT^+/+^ mice demonstrated significantly reduced bacterial levels in lungs (Figure 3A, approximate 77% mean reduction, *p* = 0.015) and in blood (Figure 3B, approximate 99% mean reduction, *p* = 0.030).

### Peripheral blood cytokine concentrations are reduced in *P.aer*-infected AAT^+/+^ mice

Excessive inflammation following bacterial infection may contribute to lung tissue damage and death (15, 43–46), and AAT possesses anti-inflammatory properties (47–53). Therefore, an AAT inhibitory effect on systemic inflammation (indicated by circulating cytokine production) may participate in the decreased mortality in AAT^+/+^ mice. We examined serum cytokine levels in six AAT^+/+^ mice and in four non-transgenic C57Bl/6 (control) mice 24 h after inhalation of *P.aer*. As depicted in Table 2 (shown under the line indicating mice infected with P.aer), infected AAT^+/+^ mice produced substantially lower blood cytokine levels than infected control animals. Cytokine reduction was numerically substantial for all tested cytokines except for IL 4, and reductions were statistically significant for 9 of the 16 cytokines tested (*p* < 0.05). In separate experiments conducted in the absence of *P.aer* infection, serum cytokine concentrations were measured in five AAT^+/+^ transgenic mice and five C57Bl/6 non-transgenic mice. The results are shown in Table 2 under the line indicating uninfected mice. Serum cytokine concentrations were uniformly low in uninfected AAT^+/+^ transgenic and C57Bl/6 non-transgenic mice.

**Table 2:** Serum cytokine levels in AAT^+/^^+^ and C57Bl/6 control mice*.

	Infected with *P.aer*			Uninfected	
	
	
Cytokine	AAT^+/+^ (*N* = 6)	C57Bl/6 (*N* = 4)	Percent reduction in infected AAT^+/+^ mice^§^	AAT^+/+^ (*N* = 5)	C57Bl/6 (*N* = 5)
IL-1α	11 ± 6.4	782 ± 668	98.6^†^	17 ± 10	65 ± 25
IL-1β	509 ± 177	4797 ± 2214	89.4^†^	0 ± 0	251 ± 138
IL-2	117 ± 50	586 ± 105	80.0^‡^	3.4 ± 3.4	10 ± 6.6
IL-3	149 ± 11	1226 ± 813	87.8^‡^	110 ± 13	92 ± 32
IL-4	88 ± 9.6	101 ± 27	12.9	110 ± 13	92 ± 32
IL-5	140 ± 89	2556 ± 344	94.5^†^	0 ± 0	0 ± 0
IL-6	2391 ± 733	12378 ± 2324	80.7^†^	11 ± 7.3	2.2 ± 1.7
IL-10	163 ± 46	529 ± 120	69.2^‡^	16 ± 7.8	11 ± 8.0
IL-12	171 ± 32	261 ± 110	34.5	17 ± 17	54 ± 28
IL-17	75 ± 11	1177 ± 607	93.6	14 ± 8.0	23 ± 13
MCP-1	464 ± 110	6920 ± 2655	93.3^†^	0 ± 0	0 ± 0
IFNγ	71 ± 7.7	1175 ± 583	94.0	30 ± 6.6	29 ± 6.2
TNFα	33 ± 6.2	400 ± 244	91.7	18 ± 6.5	18 ± 6.0
MIP-1α	157 ± 86	4812 ± 3088	96.7	17 ± 17	25 ± 13
GMCSF	115 ± 11	1321 ± 585	91.3^†^	41 ± 22	47 ± 32
RANTES	356 ± 81	3319 ± 1375	89.3	48 ± 6.4	61 ± 13

*Data expressed as means ± SEM pg/mL.

*^§^Percent reduction in AAT^+/+^ mice compared to C57Bl/6 control mice 24 h following infection*.

*^†^p = 0.01, ^‡^p = 0.02*.

### AAT does not inhibit *P.aer* bacterial growth

**Figure 4:**
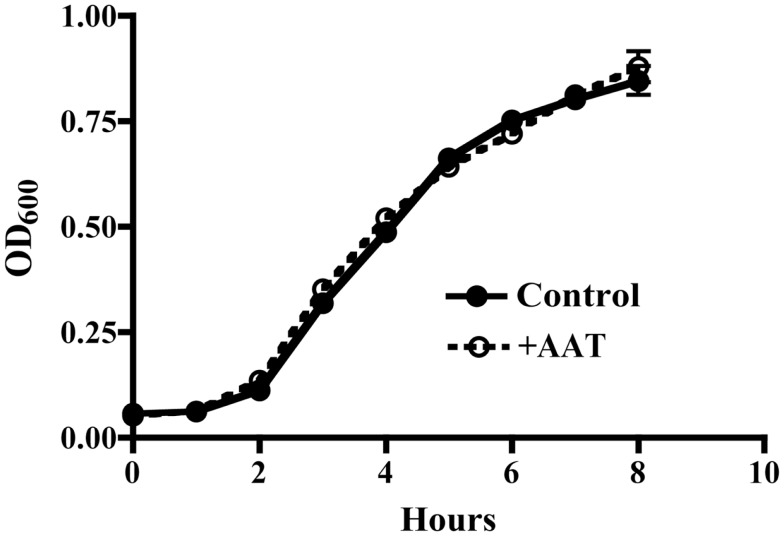
**AAT effect on *P.aer* replication in suspension cultures**. *P.aer* bacteria were inoculated into LB broth cultures in the absence (Control, closed circles with solid line) or presence of 5 mg/mL AAT (+AAT, open circles with dashed line) and incubated in a shaking incubator for 8 h at 37°C. The optical density (OD_600_) of the cultures was measured hourly. The means ± SEM OD values are shown from three separate experiments.

It is possible that AAT protective activity resulted from a direct antibacterial effect. To test this possibility, *P.aer* was grown in LB broth cultures in the absence or presence of 5 mg/mL AAT. Bacterial concentrations (OD_600_) were measured hourly to determine bacterial growth. As shown in Figure 4, AAT had no effect on bacterial growth compared to control cultures.

### AAT inhibits *P.aer* internalization into A549 lung epithelial cells, but does not inhibit bacterial adherence

**Figure 5:**
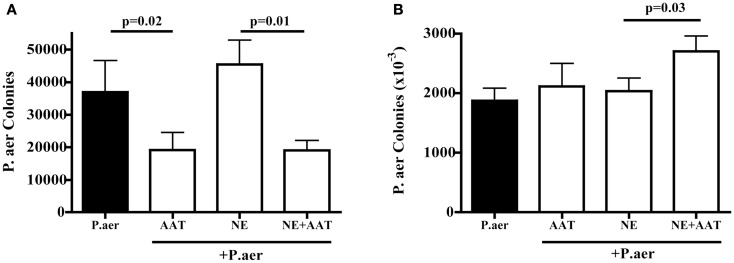
**Effect of AAT on *P.aer* internalization and adhesion in A549 cells**. In four separate experiments, A549 cells were exposed to *P.aer* alone (closed bars) or to *P.aer* in the presence of 5 mg/mL AAT, 2 μg/mL NE, or NE with AAT. In bacterial internalization experiments **(A)** and surface adhesion experiments **(B)**, A549 cells were lysed and concentrations of released bacteria calculated after culture on LB-agar plates. *p*-Values are indicated in the graphs. Data are depicted as means + SEM of bacterial colonies.

Prior studies showed that *P.aer* invades primary respiratory epithelial cells and the A549 epithelial cell line (25, 26). A549 cells were exposed to *P.aer* for 2 h, a period of time that allowed intracellular invasion of bacteria. As shown in Figure 5A, compared to *P.aer* alone (closed column), AAT significantly inhibited bacterial internalization (second column from left, mean 48% inhibition, *p* = 0.02). The effect of human NE on *P.aer* internalization into A549 lung epithelial cells was also assessed. A549 cell cultures exposed to human NE increased *P.aer* internalization by a mean 19% compared to *P.aer* alone (compare closed column and third column from left). The presence of AAT (far right column) reduced internalization levels by a mean 58% compared to NE-exposed cells (*p* = 0.01). All cultures were tested for cytotoxicity using an LDH release assay, and none was detected (not presented).

To determine if AAT inhibited bacterial association with A549 cell surfaces, A549 cells were exposed to *P.aer* in the absence or presence of AAT. Incubation for 1 h following addition of *P.aer* permitted binding of bacteria to the cell surface while minimizing intracellular invasion. As shown in Figure 5B (two bars on left), AAT did not significantly alter *P.aer* binding to the surface of A549 cells compared to cells exposed to *P.aer* alone (closed bar). We also assessed the effect of AAT on *P.aer* adherence in the presence of human NE. Figure 5B shows (third and fourth bars from left) that AAT did not reduce bacterial association with A549 cells. In fact, AAT enhanced *P.aer* association with cells incubated with human NE (*p* = 0.03). All cultures were analyzed for toxicity using an LDH release assay, and no cytotoxicity was detected (not shown).

### AAT inhibits NE- or *P.aer*-induced disruption of A549 cell monolayers

**Figure 6:**
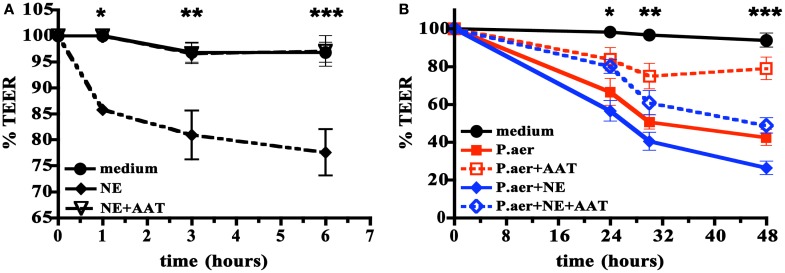
**Effect of AAT on human NE- or *P.aer*-induced disruption of A549 cell monolayers**. In **(A)** A549 cell monolayers were incubated with medium alone (medium, closed circles with solid line), human NE alone (NE, closed diamonds with dashed line), or with human NE in the presence of 5 mg/mL AAT (NE + AAT, open triangles with dashed line). Data are expressed as percent of TEER determined just prior to addition of NE (defined as *T* = 0 and set to 100%). The means ± SEM are shown for four separate experiments. *For *T* = 1 h, significant differences were noted for NE compared to medium (*p* < 0.001) and for NE compared to NE + AAT (*p* < 0.001). **For *T* = 3 h, significant differences were noted for NE compared to medium (*p* < 0.05) and for NE compared to NE + AAT (*p* < 0.05). ***For *T* = 6 h, significant differences were noted for NE compared to medium (*p* < 0.001) and for NE compared to NE + AAT (*p* < 0.001). In **(B)** A549 cell monolayers were exposed to medium alone (medium, black closed circles with solid line), *P.aer* alone (*P.aer*, red closed squares with solid line), *P.aer* with AAT (*P.aer* + AAT, red open squares with dashed line), *P.aer* and human NE (*P.aer* + NE, blue closed diamonds with solid line), or with *P.aer* and human NE with AAT (*P.aer* + NE + AAT, blue open diamonds with dashed line). Data are expressed as percent of TEER measurements in each condition just prior to addition of *P.aer* (defined as *T* = 0 and set to 100%). The means ± SEM are shown for three separate experiments. *For *T* = 24 h, significant differences were noted for medium compared to *P.aer* (*p* < 0.01), medium compared to *P.aer* + NE (*p* < 0.001), and for *P.aer* compared to *P.aer* + AAT (*p* < 0.05). **For *T* = 30 h, significant differences were noted for medium compared to *P.aer* (*p* < 0.001), medium compared to *P.aer* + NE (*p* < 0.001), *P.aer* compared to *P.aer* + AAT (*p* < 0.01), and for *P.aer* + NE compared to *P.aer* + NE + AAT (*p* < 0.05). ***For *T* = 48 h, significant differences were noted for medium compared to *P.aer* (*p* < 0.001), medium compared to *P.aer* + NE (*p* < 0.001), *P.aer* compared to *P.aer* + AAT (*p* < 0.001), and for *P.aer* + NE compared to *P.aer* + NE + AAT (*p* < 0.01).

The pathogenesis of severe pneumonia includes defects in the barrier function of respiratory epithelium. We examined the effect of human NE or viable *P.aer* on A549 cell monolayer integrity using the TEER assay, which measures the barrier integrity of cell monolayers (42). Figure 6A shows that compared to medium alone, addition of human NE to A549 cells reduced monolayer integrity by a maximum of 20% at 6 h (*p* < 0.001). Addition of 5 mg/mL AAT almost completely reversed the NE effect (*p* < 0.001, *p* < 0.05, or *p* < 0.01 comparing NE to NE + AAT at 1, 3, or 6 h, respectively).

In Figure 6B, we quantified the effect of viable *P.aer* alone or *P.aer* with human NE on A549 monolayer integrity. Cells exposed only to *P.aer* (*P.aer*) demonstrated decreased electrical resistance by a maximum 55% at 48 h compared to medium (*p* < 0.001). The presence of AAT significantly inhibited *P.aer*-induced monolayer disruption (*p* < 0.05, *p* < 0.01, or *p* < 0.001 comparing *P.aer* to *P.aer* + AAT at 24, 32, or 48 h, respectively). We also tested AAT effect in the presence of both *P.aer* and human NE. As shown in Figure 6B, the combination of *P.aer* and human NE (*P.aer* + NE) decreased monolayer resistance by 72% compared to medium at 48 h. AAT significantly reduced monolayer disruption induced by combined *P.aer* and human NE (*p* < 0.05, *p* < 0.05, or *p* < 0.01 comparing *P.aer* + NE to *P.aer* + NE + AAT at 24, 32, or 48 h, respectively). All cultures were analyzed for toxicity using an LDH release assay, and no cytotoxicity was detected (not shown).

## Discussion

New approaches are needed to reduce the clinical and economic burdens of NP. More effective NP prophylaxis would be especially welcomed, since prevention of disease or reduced disease severity can produce substantial clinical and economic benefits. Therefore, we focused on preventative (prophylactic) models of AAT use in pneumonia therapy. *P.aer* was used in our experiments since this bacterium causes up to 20% of NP cases and carries an attributable mortality of nearly 43% (2, 4, 5).

Alpha-1 antitrypsin^+/+^ mice infected with inhaled *P.aer* demonstrated nearly 100% reduced mortality compared to non-transgenic control mice (Figure 1A). This result was especially striking since the mortality in control animals was 100% and AAT^+/+^ animals did not receive antimicrobial treatment. The survival benefit in AAT^+/+^ mice was likely due to increased AAT in lungs, since human AAT transgene expression in these animals is restricted to lung tissues (40). Since transgenic AAT^+/+^ mice express human AAT constitutively, this is a model of AAT pneumonia prophylaxis.

Exogenous human AAT was tested in non-transgenic C57Bl/6 mice. AAT delivered by aerosol alone in preliminary experiments did not significantly protect mice subjected to *P.aer* pneumonia (not shown). The nebulizer we used to deliver aerosolized AAT into the airways produces 5–10 μm particles, and aerosolized droplets of 1–3 μm are required for optimal AAT distribution into peripheral airspaces (54). Therefore, we believe insufficient AAT in the peripheral airways accounted for the absence of mortality effect. We also tested AAT administration by ip injection alone in preliminary studies with the goal of increasing serum concentrations to approximately 5 mg/mL. This AAT concentration suppressed *P.aer* internalization into respiratory epithelial cells *in vitro* (Figure 5), and reduced *P.aer*-induced epithelial barrier disruption in TEER experiments (Figure 6). We determined that two ip AAT doses of 2 mg administered 24 h apart followed by a third ip AAT injection 2 h before infection increased AAT serum levels to approximately 5.0 mg/mL (not shown). However, ip-alone AAT did not improve survival in mice compared to untreated controls (not shown). Since only about 10% of the AAT concentration in the circulation is detected in extracellular lung fluid (35), the increase in lung tissue AAT following ip injection alone was insufficient to protect mice. In contrast, exogenous AAT administration using combined aerosol delivery and ip injection resulted in significant protection from *P.aer* pneumonia (Figure 1B). However, combined aerosol and ip AAT administration did not achieve the same level of protection (44% survival, Figure 1B) as observed in AAT^+/+^ transgenic mice (90% survival, Figure 1A). In future studies, we will assess *P.aer* pneumonia in non-transgenic mice using aerosol AAT delivered using a nebulizer that produces 1–3 μm droplets. We believe optimized AAT delivery to peripheral airways will enable mortality benefit using aerosol AAT monotherapy.

Histopathologic assessments of infected mouse lungs showed reduced inflammation and damage in AAT^+/+^ mice compared to non-transgenic controls (Figure 2; Table 1). Table 1 documents reduced lung pathology in AAT^+/+^ mice for all measures tested, except for increased prevalence of bronchopneumonia in AAT^+/+^ mice. Since bronchopneumonia indicates less severe lung involvement than lobar pneumonia (see Materials and Methods), this observation reflected lower pneumonia severity in AAT^+/+^ mice. Reduced percent total lung involvement was observed in AAT^+/+^ mice that approached but did not achieve statistical significance (*p* = 0.052 compared to non-transgenic controls), probably due to insufficient sample size. Reduced lung damage in AAT^+/+^ mice may represent one mechanism of improved survival in AAT^+/+^ mice.

Striking reduction in bacterial proliferation in lungs was observed in AAT^+/+^ mice (Figure 3A). AAT did not directly affect bacterial replication (Figure 4), indicating AAT antibacterial effect was indirect. Since AAT is best described as an inhibitor of the serine protease NE, AAT inhibition of NE likely participated in AAT bacterial suppression. This implies a role for NE in enhancing bacterial proliferation. NE effects that likely assist bacterial survival include proteolytic destruction of respiratory tissues and inactivation of extracellular soluble anti-pathogen substances such as immunoglobulins, complement components, defensins, lysozyme, lactoferrin, and cathelicidins (12, 13, 16, 20, 21, 39). By quenching NE proteolytic activity, AAT can promote clearance of extracellular bacteria by maintaining the structural integrity of the airways and preserving cilia-mediated bacterial evacuation. AAT-induced NE blockade can also protect the function of soluble anti-pathogen substances. A separate NE activity that can interfere with bacterial clearance is increased expression of cell-surface bacterial binding sites (MUC1) that promotes bacterial invasion into epithelial cells. Internalization shields bacteria from extracellular immune effectors such as soluble anti-pathogen substances and phagocytic cells. It is therefore noteworthy that AAT suppressed *P.aer* internalization into respiratory epithelial cells *in vitro* (Figure 5). This AAT effect may assist bacterial killing by prolonging exposure of bacteria to soluble extracellular anti-pathogen mediators and phagocytic cells (13). Taken together, we surmise enhanced *P.aer* clearance in lungs in AAT^+/+^ mice (Figure 3A) involved counteracting NE-induced airways tissue destruction, reversing NE-inactivation of soluble antibacterial substances, and blocking *P.aer* access to the interior of respiratory epithelial cells.

As shown in Figure 3B, significant reduction in the magnitude of bacteremia was also observed in AAT^+/+^ mice (99% reduction compared to non-transgenic control mice). These results suggest unrestrained NE promoted bacteremia. NE-induced proteolytic lung damage can disrupt epithelial and endothelial barrier integrity and allow translocation of bacteria from lungs into blood. NE can also disrupt epithelial barrier integrity in the absence of tissue destruction or cytolysis (Figure 6, see text below). For these reasons, AAT suppression of NE effects on tissue barrier integrity is a possible mechanism by which AAT reduced bacteremia.

Although our results implicate NE as an enhancer of bacterial survival, prior studies showed NE possesses direct antibacterial activity (55). Therefore, the inhibitory effect of AAT on bacterial proliferation (Figure 3) must be reconciled with this NE antibacterial effect. Since the reported NE antibacterial activities occur intracellularly and apply to bacteria phagocytosed by neutrophils, a detrimental AAT effect on NE-induced bacterial killing would have to occur intracellularly. However, AAT has not been shown to inhibit intracellular proteases, and AAT likely does not interfere with beneficial intracellular NE-mediated antimicrobial effects.

Systemic inflammation was profoundly reduced in infected AAT^+/+^ mice. Serum concentrations of all 16 cytokines tested in AAT^+/+^ mice were lower compared to control animals (Table 2). We believe lower circulating cytokine levels in AAT^+/+^ mice reflected attenuated severity of pneumonia in AAT^+/+^ animals. The reduced systemic inflammatory response in AAT^+/+^ transgenic animals may have contributed to improved survival following *P.aer* pneumonia.

*In vitro* studies explored mechanisms by which AAT may interrupt pneumonia pathogenesis. AAT was not directly bactericidal for *P.aer* (Figure 4), and this has been reported previously (38). *Pseudomonas* and other pneumonia-causing bacteria can invade lung epithelial cells, which may be a mechanism of immune evasion (24, 26, 56–59). Therefore, we assessed AAT for effect on bacterial invasion of respiratory epithelial cells. In our studies, AAT significantly reduced *P.aer* internalization into A549 cells (Figure 5A). Unexpectedly, we did not observe an AAT effect on *P.aer* adherence to A549 cells (Figure 5B). This suggests AAT suppression of *P.aer* internalization occurred at a post-binding step during the process of invasion. The small but statistically significant increase in *P.aer* binding to cells exposed to combined AAT and human NE (compared to infected cultures with human NE alone) is of uncertain significance (Figure 5B). Results shown in Figure 5 suggest AAT assists clearance of bacteria by blocking intracellular invasion and exposing bacteria to extracellular soluble anti-pathogen mediators and phagocytic cells. We previously reported that AAT inhibited spontaneous internalization of *Mycobacterium abscessus* into human monocyte-derived macrophages (60). These parallel results raise the possibility that an unappreciated AAT function is to deny access of pathogens to the intracellular compartment. It is also noteworthy that AAT reduced spontaneous (absence of human NE) *P.aer* invasion of A549 cells (second bar from left in Figure 5A), indicating that AAT can block invasion using a mechanism unrelated to NE inhibition. Other reports also suggest AAT possesses activities independent of serine protease inhibition. These include suppression of cytokine production in whole blood or in monocytes, and inhibition of HIV replication (50, 61–66).

Respiratory epithelial barrier dysfunction is a pivotal component of severe pneumonia that can result in impaired gas exchange, seepage of fluid into the respiratory tract, and decreased bacterial containment with ensuing bacteremia (46). In TEER experiments in A549 cell monolayers, human NE, *P.aer*, or combined NE and *P.aer* significantly reduced monolayer barrier integrity (Figure 6). AAT reduced monolayer disruption induced by human NE, *P.aer*, or combined human NE and *P.aer* (Figures 6A,B). Therefore, one mechanism of AAT protection against pneumonia-related pathology is suppressed disruption of the pulmonary epithelium barrier. No cytotoxicity was observed in the TEER experiments (not shown), suggesting monolayer alterations involved changes in the integrity of intercellular junctions. Prior reports showed *P.aer* or LPS disrupted respiratory cell barrier function, and altered intercellular junction integrity accompanied these effects (67, 68). We speculate that AAT prevents epithelial barrier disruption *in vivo* by blocking alterations in intercellular tight junctions induced during inflammation and infection.

Our studies suggest that AAT is an endogenous molecule with pneumonia-suppressive function, which implies that AAT deficit weakens host defense against pneumonia. In a study of deaths in persons with genetic AAT deficiency, Tomashefski et al. (69) noted that mortality due to pneumonia approached 37%, the highest immediate cause of death in these patients. In comparison, pneumonia (including influenza) was the cause of death in 2.2% of the US population in 2009 (70). The extraordinary mortality caused by pneumonia in AAT deficient persons suggests predisposition to severe pneumonia due to AAT deficit. Similarly, studies in patients without known AAT deficiency suggest association between a deficit in AAT function in lung tissues and pneumonia. Braun et al. (71) measured BALF AAT levels in patients with acute pneumonia. Although BALF AAT concentrations were higher in pneumonia patients than in healthy controls, the serine protease inhibitor function of AAT was reduced in pneumonia patients. The authors surmised that proteases and reactive oxygen species (produced by neutrophils and lung epithelia) degraded AAT serine protease inhibitor function in the airways. Taken together, these reports document association between reduced AAT levels or reduced AAT function and pneumonia, and suggest a pneumonia-suppressive role for AAT. A pneumonia-suppressive role for AAT implies AAT augmentation should reduce frequency or severity of pneumonia. In fact, two reports suggest this is the case. Lieberman examined the self-reported incidence of lung infections in patients with genetic AAT deficiency, and noted three to five lung infections per year in AAT deficient patients not receiving AAT replacement therapy (72). In contrast, only zero to one infections per year were described in AAT deficient patients receiving intravenous AAT replacement therapy. In a rat model of chronic *P.aer* lung infection, aerosol delivery of AAT decreased lung inflammation and accelerated bacterial clearance (38).

## Conclusion

Several limitations apply to our studies. We examined AAT effects as pneumonia prophylaxis (AAT present before onset of pneumonia), and we did not evaluate AAT administration as a treatment after infection. Although we believe AAT administration will be beneficial after onset of pneumonia, AAT effect as a non-preventative therapy cannot be confidently inferred from our results. Also, the generalizability of the AAT anti-pneumonia effect to bacteria other than *P.aer* is uncertain. Technical limitations likely prevented us from obtaining mortality reduction in mice treated with exogenous AAT equivalent to mortality reduction in AAT^+/+^ mice (Figure 1). Optimal AAT deposition in peripheral lung tissues requires aerosol droplets of 1–3 μm, and the nebulizer used in our studies produced particle sizes of 5–10 μm. Additionally, our *in vitro* pathogenesis studies were conducted in the A549 respiratory epithelial cell line, and cell lines do not necessarily reflect characteristics of primary cells *in vivo*. The AAT concentration used in our *in vitro* studies (5 mg/mL) is consistent with serum levels attained during acute disease. However, AAT concentrations in the lung microenvironment that enable AAT antibacterial effects are unknown. This introduces some uncertainty regarding the relevance of our *in vitro* mechanistic studies to our *in vivo* results. On the other hand, since augmented AAT levels in the respiratory tract enhanced survival (Figure 1), increasing AAT biologic activity sufficient to confer benefit *in vivo* is possible. Finally, although animal pneumonia models are useful, projecting our results to efficacy in humans entails risk.

In this report, we show protective AAT effects in mice with experimentally induced acute *P.aer* pneumonia. AAT expression in the lungs of AAT^+/+^ mice was associated with reduced pneumonia mortality, lower severity of lung inflammation, decreased bacterial proliferation in lungs, prevention of bacteremia, and suppressed systemic inflammatory response. *In vitro* studies demonstrated AAT blockade of *P.aer* internalization into lung epithelial cells and AAT suppression of NE- or *P.aer*-induced epithelial cell barrier disruption. AAT did not demonstrate direct antibacterial activity, and AAT protection likely involved both NE neutralization and effects independent of NE neutralization. We believe AAT protection in mouse pneumonia (Figure 1) was due to inhibition of NE-induced proteolytic lung damage, reduction of NE lysis of extracellular anti-pathogen immune mediators, blockade of *P.aer* invasion of respiratory epithelial cells, and suppression of epithelial barrier dysfunction. Our studies are different from those reported by Cantin and Woods (38). Although our results and those in the Cantin and Woods report show AAT reduced bacterial density in lungs, Cantin and Woods studied chronic *Pseudomonas* lung infection in rats. Also, aerosol AAT was administered daily for 7 days after infection. The outcomes in Cantin and Woods included reduced lung bacteria and lung inflammation 7 days post-AAT initiation, and reduced airway NE activity 6 h after onset of AAT administration.

Alpha-1 antitrypsin appears to possess broad-spectrum anti-pathogen function, since AAT at physiological concentrations demonstrated inhibitory effects *in vitro* against *Mycobacterium abscessus* and HIV (64, 73,75). In fact, an AAT-derived synthetic molecule suppressed HIV replication following intravenous infusion into infected patients (66). Given these observations, we speculate that AAT is an innate immune mediator. It appears that AAT blocks activity of host molecules required for pathogen replication (such as NE) and AAT counteracts pathogen evasion of host immune defenses. Since extracellular lung fluid levels of AAT are only about 10% of serum concentrations (35), the lung may represent an “Achilles heel” of AAT immune protection. This observation provides rationale for using inhaled AAT for prophylaxis or treatment of respiratory tract infections. Results in this report suggest that clinical application may include AAT use as an inhaled drug for NP prophylaxis. Combination treatment using inhaled and intravenous AAT delivery may provide additional protection. Since AAT does not target pathogen-specific molecules, AAT antimicrobial effects may be impervious to genetic mutations that alter pathogen components. Furthermore, since AAT has been available for clinical use since 1988 and AAT has an impressive record of safe use in humans, application of AAT for this indication can proceed rapidly to the bedside (37, 76, 77).

## Conflict of Interest Statement

Leland Shapiro is a shareholder of Omni Bio Pharmaceutical, Inc. All other authors declare that the research was conducted in the absence of any commercial or financial relationships that could be construed as a potential conflict of interest.
